# Biodegradable Temporizing Matrix for Facial Reconstruction Following Mohs Micrographic Surgery: A Multi-Subunit Analysis

**DOI:** 10.3390/bioengineering13070732

**Published:** 2026-06-24

**Authors:** Alexandra L. Martinez, James F. Thornton

**Affiliations:** Department of Plastic Surgery, UT Southwestern Medical Center, Dallas, TX 75390, USA; james.thornton@utsouthwestern.edu

**Keywords:** biodegradable temporizing matrix, facial reconstruction, Mohs micrographic surgery, dermal substitute, nasal reconstruction, periocular reconstruction, scalp reconstruction, wound healing, tissue engineering

## Abstract

Background: Biodegradable Temporizing Matrix (BTM) is a synthetic, biodegradable polyurethane dermal substitute that promotes neodermis formation and supports re-epithelialization by secondary intention. However, published experience with BTM in facial reconstruction is limited to small case series with narrow anatomic indications, and its performance across multiple distinct facial subunits has not been systematically characterized. Methods: A retrospective case series was conducted of all patients who underwent nasal, periocular, or scalp reconstruction with BTM following Mohs micrographic surgery (MMS) at a single tertiary academic center. Patient demographics, defect characteristics, healing times, complications, and secondary procedures were recorded. Results: A total of 135 patients were included: 67 nasal, 40 periocular, and 28 scalp. Overall complication rates were 7.5%, 20.0%, and 17.9%, respectively. The majority of patients across all sites achieved definitive coverage by secondary intention without skin grafting. Lower eyelid involvement was the strongest independent predictor of ocular complications in the periocular cohort, and prior radiation was the dominant predictor of scalp complications. Conclusions: In this retrospective series, BTM demonstrated favorable outcomes for facial reconstruction following MMS across nasal, periocular, and scalp subunits, reliably supporting secondary re-epithelialization and functioning as definitive reconstruction, a temporizing agent, and a lining adjunct for staged forehead flap reconstruction. Prospective comparative studies are needed to confirm these findings.

## 1. Introduction

The head and neck represent the most common sites for cutaneous malignancy, and Mohs micrographic surgery (MMS) remains the gold standard for treatment, offering maximal tissue preservation with the highest cure rates [1,2,3,4]. The resulting defects vary considerably in size, depth, and anatomic complexity, and reconstruction of the face presents challenges that are not encountered elsewhere on the body. The nose, scalp, and periocular region are among the most technically demanding areas for post-Mohs reconstruction, each imposing distinct constraints with respect to functional preservation, aesthetic outcome, and tissue availability [5,6].

Advances in biomedical engineering have expanded reconstructive options through the development of acellular dermal substitutes that promote neodermis formation and broaden the surgical toolkit for complex facial defects. NovoSorb^®^ Biodegradable Temporizing Matrix (BTM; PolyNovo Biomaterials Pty Ltd., Port Melbourne, Australia) is a fully synthetic bilayer composed of biodegradable polyurethane, consisting of an outer sealing membrane that limits moisture loss and bacterial infiltration, and an inner open-cell matrix that serves as a scaffold for neodermis ingrowth [7,8,9,10,11]. Originally developed for major burn reconstruction, BTM has demonstrated broad applicability across surgical wounds, trauma defects, and oncologic resection sites. Critically, BTM supports re-epithelialization by secondary intention without requiring a mandatory secondary skin grafting procedure, a property that simplifies the reconstructive pathway for many patients [12,13,14].

Despite its expanding clinical use, published experience with BTM in facial reconstruction remains limited to small case series and reports focused on narrow subunit indications [14,15,16,17]. No study has evaluated BTM comprehensively across multiple distinct facial subunits in a single cohort. This knowledge gap is clinically important, as each facial region—nose, scalp, and periocular area—presents distinct reconstructive demands, and the utility of BTM may differ accordingly. This study presents the largest multi-subunit evaluation of BTM for facial reconstruction following MMS to date. The objectives are: (1) to characterize clinical outcomes with BTM across nasal, periocular, and scalp reconstruction; (2) to identify site-specific risk factors and subunit-level considerations; and (3) to propose updated reconstructive algorithms incorporating BTM for each anatomic region.

## 2. Materials and Methods

### 2.1. Study Design and Patient Population

This study was approved by the UT Southwestern Medical Center Institutional Review Board. A retrospective review was conducted of all consecutive patients who underwent nasal, periocular, or scalp reconstruction with BTM following MMS by a single surgeon at a single tertiary academic hospital from May 2019 to January 2026. Inclusion criteria were: (1) Mohs micrographic surgery defect of the nose, periocular region, or scalp; (2) reconstruction with BTM as the primary wound management strategy; and (3) minimum one postoperative follow-up visit documenting wound status. Exclusion criteria were: (1) incomplete medical records and (2) loss to follow-up prior to documentation of wound outcome. BTM was selected over local flaps or skin grafting at the surgeon’s discretion based on defect size and depth, patient comorbidities, patient preference to avoid additional donor sites or flap procedures, and anatomic constraints limiting local tissue availability. BTM was also selected in patients undergoing staged oncologic clearance in whom temporizing reconstruction was desired. Patients with incomplete follow-up were excluded. Patient demographics, medical history, defect characteristics, operative details, healing times, complications, and secondary procedures were recorded for each patient.

### 2.2. Outcome Measures

Primary outcomes included: time to sealing membrane delamination (defined as complete BTM integration with neodermis formation, at which point the outer sealing membrane was manually removed by the surgeon); time to definitive coverage (defined as complete healing of the surgical site); overall complication rate; and rate of secondary surgical procedures. Site-specific complications were tracked. For the periocular cohort, ocular and eyelid complications including ectropion, retraction, lagophthalmos, ptosis, canthal dystopia, and exposure keratoconjunctivitis were reported. For the scalp cohort, delayed healing, wound breakdown, and persistent bone exposure were reported. For the nasal cohort, infection and delayed healing were reported. Revision procedures and in-office aesthetic refinements were recorded separately.

### 2.3. Statistical Analysis

Outcomes were analyzed descriptively for the nasal cohort. For the periocular and scalp cohorts, multivariable logistic regression was used to identify independent predictors of binary outcomes (complications, secondary procedures), and multivariable linear regression was used for continuous outcomes (healing times). All analyses were performed using R (version 4.5.2). Continuous variables are reported as means ± standard deviations (SD) or medians with interquartile ranges (IQR). Categorical variables are reported as frequencies and percentages. Statistical significance was defined as *p* < 0.05. Given the relatively small sample sizes of the periocular (*n* = 40) and scalp ((*n*= 28) cohorts, multivariable models were limited to a small number of covariates to minimize overfitting risk. Confidence intervals are reported where available; wide confidence intervals and large odds ratios should be interpreted with caution given the limited statistical power of these analyses.

### 2.4. Surgical Technique

Prior to BTM placement, the wound bed was debrided of all non-viable tissue to achieve a uniformly vascular substrate. For scalp defects with exposed calvarium, a water-cooled diamond cutting burr was used to create punctate cortical bleeding, which is essential for matrix integration over avascular bone [13]. For nasal defects, a template was created by pressing the BTM sheet against the wound to conform to its geometry prior to trimming. The BTM was trimmed to the defect dimensions and inset with Monocryl sutures at the wound periphery, with Monocryl or Prolene tacking sutures placed centrally to ensure full-surface contact and prevent shearing or wrinkling of the sheet.

Following inset, mupirocin ointment was applied to the sheet surface and covered with a non-stick dressing. No bolster dressing was required. The dressing was removed in clinic at 4–7 days. Patients were instructed to keep the site dry for 7 days, after which showering was permitted. Sealing membrane removal was performed once the BTM had fully integrated, identified by uniform pink coloration of the construct, typically within 3–5 weeks. The wound bed was then allowed to complete epithelialization by secondary intention, or a skin graft was applied when clinically indicated.

## 3. Results

### 3.1. Nasal Reconstruction

The nasal cohort included 67 patients (50.7% male; mean age 70.4 ± 13.8 years; mean follow-up 3.2 months). Mean defect size was 4.68 ± 4.25 cm^2^. The most common defect etiology was basal cell carcinoma (BCC; 73.1%). The most commonly involved nasal subunits were the ala (47.8%) and tip (47.8%). Twelve patients (17.9%) had nasal lining defects.

**Figure 1:**
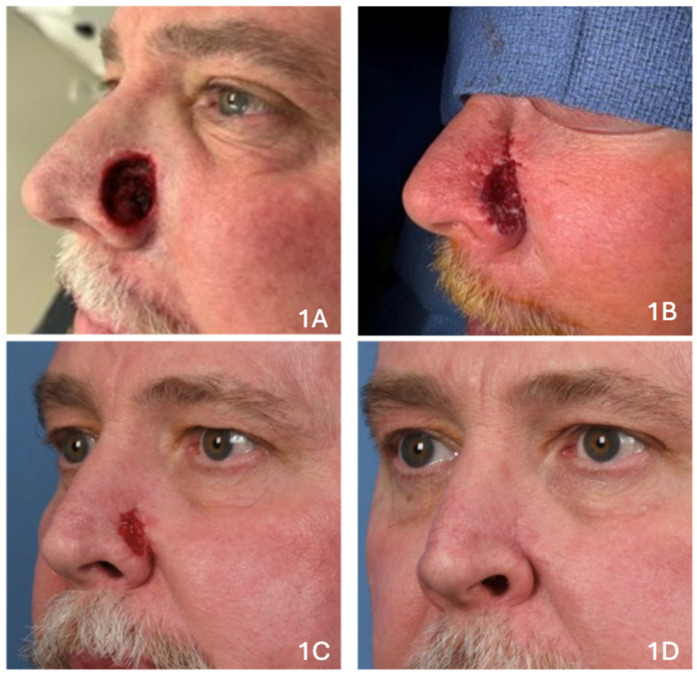
Healing stages of BTM placement on a patient with a nasal defect of his left sidewall and ala. (**A**) Nasal defect immediately after Mohs surgery. (**B**) Nasal defect immediately after application of BTM. (**C**) Patient 28 days after initial BTM placement. BTM was fully integrated, and the patient is shown after the sealing membrane was delaminated. (**D**) Nasal defect completely epithelialized.

The mean time to sealing membrane delamination was 35.2 ± 14.9 days, and the mean time to definitive coverage was 69.7 ± 30.1 days. The overall complication rate was 7.46%, comprising one infection (1.49%) and four cases of delayed healing (5.97%); two of the delayed healing patients healed secondarily and two received full-thickness skin grafts (FTSG). Twelve patients (17.9%) underwent a secondary surgical procedure: six planned paramedian forehead flaps, five FTSGs (including the two delayed healing cases), and one composite graft. Thirteen patients (19.4%) underwent in-office aesthetic refinement including dermabrasion (38.5%), microneedling (30.8%), and triamcinolone acetonide injection (30.8%). The majority of patients achieved definitive coverage by secondary intention without skin grafting. Nasal outcomes are summarized in Table 1. See Figure 1 for an example of BTM used in nasal reconstruction.

### 3.2. Periocular Reconstruction

The periocular BTM cohort included 40 patients (62.5% male; mean age 77.5 years; median follow-up 2.3 months, IQR 1.2–5.5). Median defect size was 10.2 cm^2^ (IQR 3.3–27.5). The most common defect etiologies were BCC (41.4%), melanoma (29.7%), and squamous cell carcinoma (SCC; 28.1%). The most frequently involved subunits were the cheek (26.1%), temple (19.9%), nasal sidewall (19.5%), and lower eyelid (12.4%). Approximately one third of patients had more than one subunit involved.

**Figure 2:**
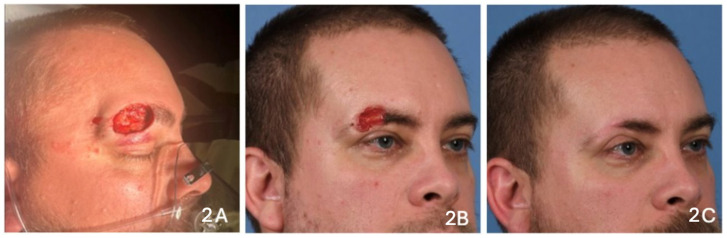
(**A**) A 44-year-old male presented with a 12 cm^2^ defect of the right eyebrow and upper eyelid after MMS for SCC. (**B**) Thirty days after reconstruction with BTM. (**C**) Nine months after reconstruction, the patient’s defect healed with an adequate color and contour match. Hair transplantation is an option for patients after reconstruction with BTM.

The overall complication rate was 20.0% (8/40). Ocular complications occurred in 15.0% of patients (6/40) and included retraction (7.5%), ectropion (2.5%), lagophthalmos (2.5%), ptosis (7.5%), and canthal dystopia (2.5%). Wound complications occurred in 5.0% of patients (2/40). Four patients (10.0%) required additional surgical procedures, and four patients (10.0%) required revision surgery. Median time to sealing membrane delamination was 38 days (IQR 34–46), and median time to definitive coverage was 64 days (IQR 52–77.5). The majority of patients achieved definitive coverage without secondary skin grafting. On multivariable logistic regression, lower eyelid involvement was the strongest independent predictor of any complication (OR 6.96, 95% CI 1.92–25.18; *p* = 0.003) and any ocular complication (OR 9.49, 95% CI 2.54–35.47; *p* < 0.001). Cheek involvement was additionally associated with both outcomes. Periocular BTM outcomes are summarized in Table 1. See Figure 2 for an example of BTM used in periocular reconstruction.

### 3.3. Scalp Reconstruction

The scalp BTM cohort included 28 patients (61.2% male; mean age 73.9 ± 13.2 years). Mean defect size was 31.58 ± 21.77 cm^2^. The most common etiology was SCC (61.2%), followed by melanoma (16.3%) and BCC (14.3%). Exposed calvarium was present in 71.4% of patients; bone burring was performed in 73.5% of the overall scalp cohort. A history of prior radiation and/or chemotherapy was present in 22.4% of patients.

**Figure 3:**
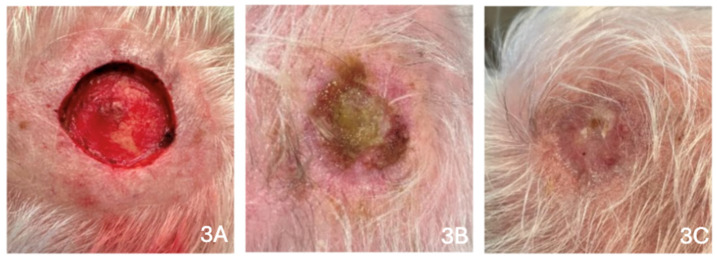
(**A**) A patient’s parietal scalp defect after MMS for melanoma in situ. (**B**) Fifty-three days after BTM placement, the sheet was delaminated. (**C**) Seventy-four days after BTM placement, the scalp defect was healed.

Mean time to sealing membrane delamination was 45.6 ± 18.9 days, and mean time to definitive coverage was 110.1 ± 91.7 days. The overall complication rate was 17.9% (5/28), with complications including delayed healing, wound breakdown, and persistent bone exposure. Five patients (17.9%) required secondary surgical procedures; only one (3.6%) required a secondary split-thickness skin graft (STSG). On multivariable logistic regression, prior radiation was the strongest independent predictor of complications (OR 22.48; *p* = 0.04) and showed a trend toward predicting secondary procedures (OR 14.66; *p* = 0.055). Exposed bone demonstrated a non-significant trend toward increased complication risk (OR 8.43; *p* = 0.087). Scalp BTM outcomes are summarized in Table 1. See Figure 3 for an example of BTM used in scalp reconstruction.

## 4. Discussion

### 4.1. BTM as a Bioengineered Platform for Facial Reconstruction

This study presents the most comprehensive evaluation of BTM for facial reconstruction to date, characterizing outcomes across three distinct facial subunits in a single institutional series of 135 patients. The findings demonstrate that BTM demonstrated favorable outcomes across all three anatomic sites, with low to acceptable complication rates, reliable secondary re-epithelialization, and manageable rates of secondary procedures in this single-surgeon retrospective series. Across the nasal, periocular, and scalp cohorts, the majority of patients achieved definitive wound coverage without skin grafting, underscoring one of BTM’s most clinically valuable properties as a bioengineered construct.

The mechanism of action of BTM derives from its open-cell polyurethane foam architecture, which modulates the wound healing environment in a manner distinct from that of biologic dermal substitutes. By compartmentalizing fibroblast activity into discrete interconnected units, BTM limits the transdifferentiation of fibroblasts to myofibroblasts and disrupts contiguous collagen architecture, thereby attenuating wound contraction and scar burden [18,19]. This anti-contractile property is particularly relevant in the facial context, where cicatricial forces can cause functional deformity—most notably ectropion in the periocular region and alar retraction at the nose. BTM’s synthetic composition additionally eliminates the risk of xenogeneic immune reactions, and its fully biodegradable chemistry avoids the need for removal once integration is complete [7,20].

A key clinical advantage of BTM across all three sites in this series is its capacity to support re-epithelialization by secondary intention, thereby enabling single-stage reconstruction in most patients [14]. This property eliminates the mandatory second-stage skin grafting required with alternative biologic matrices, thus reducing operative burden, shortening time to definitive coverage, and eliminating donor site morbidity. The postoperative simplicity of BTM is an additional practical advantage: the absence of a bolster requirement reduces patient discomfort, eliminates bolster removal visits, and decreases resource utilization. Together, these properties establish BTM as a compelling bioengineered option within the facial reconstructive toolkit.

### 4.2. Nasal Reconstruction

The nose is the most prominent aesthetic feature of the face and is uniquely challenging to reconstruct due to its complex curvatures, variation in skin thickness across subunits, and the frequent need to restore multiple structural layers [6,21]. The subunit principle guides nasal reconstruction, with local flaps and the paramedian forehead flap serving as the standard approaches for larger or full-thickness defects [22,23]. BTM serves three complementary roles within this framework.

First, for small to moderate skin-only defects (particularly of the ala and tip, where bilobed flap reconstruction is commonly considered), BTM allows reliable healing by secondary intention with favorable contour preservation. Unlike bilobed flaps, BTM avoids complex incision lines and the risks of alar retraction or pincushion deformity [24,25], while augmenting soft tissue volume without additional incisions and yielding a color match that reliably approximates adjacent nasal skin. The complication rate of 7.46% in this series is comparable to published rates for full-thickness skin grafts and other dermal substitutes in nasal reconstruction [14,26,27], and the low infection rate of 1.49% is consistent with BTM’s reported infection resilience in published clinical series [20,28]. The resistance of BTM to clinical infection is thought to be conferred in part by its outer sealing membrane, which physically limits bacterial infiltration into the wound bed, as well as by the isolated microenvironments created by its open-cell foam architecture, which may limit bacterial dissemination. However, we acknowledge that formal evaluation of bacterial biofilm behavior on the polyurethane surface of BTM—a question with relevance to long-term implant safety—was not performed in this study, and the mechanistic basis of BTM’s infection resistance has not been fully characterized at the microbiological level. This represents an important area for future basic science investigation.

Second, BTM functions as a reliable temporizing agent in the oncologic setting, protecting exposed cartilage and soft tissue while preventing wound contraction and distortion of nasal landmarks during the interval between MMS and final margin clearance. Third, when a staged paramedian forehead flap is planned, BTM applied to nasal lining defects can be re-elevated and turned inward as an intrinsic lining source during the initial flap harvest, obviating the need for separate lining procedures such as turn-in flaps or composite grafts. The 19.4% in-office aesthetic refinement rate in this cohort is comparable to published revision rates following Mohs defect repairs [29] and should be incorporated into preoperative patient counseling.

### 4.3. Periocular Reconstruction

The periocular region presents unique reconstructive demands stemming from the convergence of multiple aesthetic subunits adjacent to critical functional structures, including the eyelid margin, lacrimal system, and medial canthus [4,30]. Ectropion is the most functionally significant complication following periocular reconstruction, threatening ocular surface integrity and vision. In this series, ectropion occurred in only 2.5% of BTM patients, which compares favorably with published ectropion rates of 10–20% reported with Integra Dermal Regeneration Template in the periocular region [30]. While direct head-to-head comparisons are not available in the present series, this contrast in contextual rates is consistent with the distinct anti-contractile properties of BTM’s synthetic polyurethane architecture relative to biologic collagen-based matrices, which undergo more pronounced contraction during remodeling. Unlike Integra, BTM does not require a mandatory staged skin grafting procedure and does not carry the risk of xenogeneic immune response; relative to AlloDerm and Matriderm, BTM offers the additional advantage of a closed outer sealing membrane that maintains a moist wound environment and limits bacterial infiltration without requiring bolster fixation [19,20]. BTM’s anti-contractile mechanism, which limits myofibroblast-driven cicatricial forces, is the likely explanation for this favorable outcome, particularly in the lower eyelid where gravitational and contractile forces act in concert [18,19].

Multivariable regression in this cohort identified lower eyelid involvement as the strongest independent predictor of both any complication (OR 6.96; *p* = 0.003) and any ocular complication (OR 9.49; *p* < 0.001), while cheek involvement contributed additional risk. Reassuringly, the remaining periocular subunits (temple, medial canthus, nasal sidewall, upper eyelid, and eyebrow) did not independently predict complications, supporting the safety of BTM reconstruction in these regions. The 10.0% additional surgery rate in this cohort reflects our practice of allowing BTM to re-epithelialize by secondary intention, which avoids a mandatory grafting stage in the majority of patients. When a dermal substitute is indicated for periocular reconstruction, BTM represents a favorable option in our experience given its low ectropion risk, single-stage potential, and simplified postoperative management; however, direct comparative data with other matrices are lacking, and surgeon judgment and patient factors should guide selection. Lower eyelid defects warrant particular attention during counseling given the anatomic vulnerability of this subunit.

### 4.4. Scalp Reconstruction

Scalp defects present challenges unique among facial sites, including the frequent presence of exposed calvarium, the scalp’s limited tissue mobility, and the high prevalence of prior radiation in this population. BTM performed reliably in this setting, with a complication rate of 17.9% and a secondary STSG rate of only 3.6%, the latter representing a clinically meaningful advantage given that secondary grafting adds operative morbidity and donor site burden. The ability of BTM to support re-epithelialization over prepared bone, when cortical drilling is performed to induce punctate bleeding, extends its applicability even to defects with absent periosteum [12].

The most notable finding in the scalp cohort is the substantial impact of prior radiation on outcomes: irradiated patients had approximately 22-fold higher odds of complications (OR 22.48; *p* = 0.04). These findings have direct clinical implications: patients with a history of scalp irradiation should receive thorough preoperative counseling about significantly elevated healing risks, and close postoperative monitoring is warranted regardless of defect size or choice of matrix. Adjunctive measures such as hyperbaric oxygen therapy may be considered in this population. Exposed bone demonstrated a non-significant trend toward increased complications (OR 8.43; *p* = 0.087), consistent with the technical challenges of matrix integration over avascular calvarium and underscoring the importance of meticulous bone preparation. Defect size and smoking were not significant predictors, suggesting that local tissue factors carry more weight than systemic variables in determining scalp BTM outcomes.

An important practical advantage of BTM in scalp reconstruction is the preservation of future hair transplantation options. Split-thickness skin grafts, which lack hair follicles, preclude transplantation, whereas the neodermis generated by BTM is amenable to subsequent follicular unit transplantation [31,32,33]. This consideration is particularly relevant for younger patients or those with scalp defects in cosmetically prominent locations.

### 4.5. Limitations

This study has several limitations. The retrospective, single-surgeon design limits generalizability and introduces potential selection bias in patient and defect characteristics across cohorts. Explicit inclusion and exclusion criteria were not prospectively defined; all consecutive patients who underwent BTM reconstruction by the senior author over the study period were included, and patients with incomplete follow-up were excluded, which may introduce attrition bias. Long-term aesthetic outcomes and patient-reported satisfaction were not systematically captured. Objective scar quality metrics were not collected. Follow-up duration was relatively short across all cohorts (nasal: mean 3.2 months; periocular: median 2.3 months; scalp: not separately reported), which is insufficient to characterize scar maturation, delayed contracture, long-term ectropion, pigmentary change, or contour stability, all of which may evolve over 12–18 months following facial reconstruction. The outcomes reported here therefore represent early postoperative findings rather than definitive long-term results, and this distinction should be considered when interpreting the conclusions. The absence of a comparator group (e.g., biologic dermal substitutes, skin grafts, or local flaps) precludes direct conclusions regarding the superiority of BTM, and descriptors such as “safe and effective” should be understood in the context of this uncontrolled case series rather than as claims of comparative efficacy. The periocular and scalp cohorts were relatively small, which may limit the statistical power of multivariable regression analyses and increase the risk of overfitting; the large odds ratios observed (particularly for prior radiation) should be interpreted cautiously and confirmed in larger prospective series. Although the anti-contractile mechanism of BTM has been described in the literature, the present study does not include direct mechanistic data (e.g., myofibroblast quantification or scar histology) to independently corroborate this proposed mechanism in the facial context. Future prospective, multicenter studies with standardized outcome measures, longer follow-up, patient-reported outcome instruments, validated scar assessment tools, and comparative arms are needed to validate these findings and further define the optimal role of BTM across facial subunits.

## 5. Conclusions

This retrospective, single-surgeon case series reports favorable early outcomes with BTM for facial reconstruction following Mohs micrographic surgery across nasal, periocular, and scalp subunits. Across all three sites, BTM supported re-epithelialization by secondary intention in the majority of patients, with low to acceptable complication rates and limited need for secondary skin grafting. Site-specific risk factors were identified: lower eyelid involvement was the dominant predictor of complications in the periocular cohort, and prior radiation was the strongest predictor in the scalp cohort. These findings should be interpreted in the context of this study’s inherent limitations, including the absence of a control group, short follow-up duration, and small cohort sizes for multivariable analysis. The conclusions support BTM as a clinically useful option within the reconstructive toolkit for select facial defects following MMS, while underscoring the need for prospective comparative studies with longer follow-up and standardized outcome measures to more definitively characterize its role.

Updated reconstructive algorithms incorporating BTM are proposed for each site. For nasal defects, BTM serves as definitive reconstruction for skin-only defects, as a temporizing agent during oncologic clearance, and as a lining adjunct for staged forehead flap reconstruction. For periocular defects, BTM is the preferred dermal substitute when primary closure or local flap is not feasible, with particular emphasis on its use in lower eyelid defects given its low ectropion risk. For scalp defects with intact periosteum, BTM is an excellent first-line option; patients with prior radiation require heightened counseling regardless of matrix selection. These findings establish BTM as a foundational tool in the biomedical engineering of facial reconstruction and support further prospective investigation into its expanding role.

## Figures and Tables

**Table 1:** BTM Outcomes by Anatomic Site.

	Nasal (*n* = 67)	Periocular (*n* = 40)	Scalp (*n* = 28)
Time to membrane delamination, days	35.2 ± 14.9	38 (IQR 34–46) ^†^	45.6 ± 18.9
Time to definitive coverage, days	69.7 ± 30.1	64 (IQR 52–77.5) ^†^	110.1 ± 91.7
Overall complication rate	5 (7.5%)	8 (20.0%)	5 (17.9%)
Secondary surgical procedure rate	12 (17.9%)	4 (10.0%)	5 (17.9%)
Secondary skin graft rate	2 (3.0%) *	0 (0%)	1 (3.6%)

Values are mean ± SD unless otherwise noted. * Includes 2 FTSG for delayed healing. ^†^ Reported as median (IQR) due to non-normal distribution. FTSG = full-thickness skin graft; IQR = interquartile range; SD = standard deviation.

## Data Availability

The data presented in this study are available on request from the corresponding author. The data are not publicly available due to patient privacy restrictions.

## Abbreviations

The following abbreviations are used in this manuscript:

MMS	Mohs micrographic surgery
BTM	Biodegradable Temporizing Matrix
BCC	Basal cell carcinoma
SCC	Squamous cell carcinoma
FTSG	Full-thickness skin graft
STSG	Split-thickness skin graft
OR	Odds ratio
CI	Confidence interval
IQR	Interquartile range
SD	Standard deviation
